# A Rare Case of Uterine Blastomycosis and Its Management: A Case Report and Literature Review

**DOI:** 10.7759/cureus.52252

**Published:** 2024-01-14

**Authors:** Akbar Hussain, Fares Khater, Ali Raza, Huzefa Bhopalwala, Jonathan Piercy

**Affiliations:** 1 Internal Medicine, Appalachian Regional Healthcare, Harlan, USA; 2 Internal Medicine, Appalachian Regional Healthcare, Pikeville, USA; 3 Internal Medicine, Appalachian Regional Healthcare, Whitesburg, USA; 4 Cardiovascular Medicine, Mayo Clinic, Rochester, USA

**Keywords:** extrapulmonary complications, immunocompromised hosts, pulmonary infection, rare systemic infection, antifungal treatment, uterine blastomycosis

## Abstract

The report delineates the rare occurrence of uterine blastomycosis, an atypical systemic presentation of *Blastomyces dermatitidis* infection prevalent in North America. Focused on a 51-year-old immunocompetent female displaying abdominal pain and irregular vaginal bleeding, it underscores the intricate diagnostic hurdles posed by symptoms mirroring common gynecological conditions. Despite fewer than 10 recorded cases, the rarity of uterine involvement highlights the imperative for heightened clinical suspicion. The multifaceted diagnostic strategy integrates risk factors, travel history, imaging, and histopathological examinations. Emphasizing a multidisciplinary treatment helmed by gynecologists, pathologists, and infectious disease specialists, the utilization of antifungal agents, notably itraconazole, is pivotal. Addressing the scarcity of literature and the condition's clinical resemblance to prevalent ailments, further research becomes paramount in devising tailored diagnostic and treatment protocols for uterine blastomycosis. This study enriches the existing literature by providing critical insights into a scarcely documented condition, contributing novel perspectives essential for clinical understanding and management strategies.

## Introduction

Blastomycosis, stemming from the dimorphic fungus *Blastomyces dermatitidis*, typically presents as a pulmonary infection upon inhaling fungal spores [1]. Although endemic to North America, particularly in regions like the Great Lakes and Mississippi Valley, rare systemic manifestations have been documented [2-3]. Immunocompetent hosts often exhibit asymptomatic infection, while immunocompromised individuals face heightened morbidity and mortality, necessitating vigilant clinical suspicion for prompt diagnosis and treatment [4]. Pulmonary complications involve pneumonia, while systemic implications encompass skin, bone, genitourinary tract, and central nervous system dissemination [5-6]. *Blastomyces dermatitidis*' dimorphic nature, transitioning from mold to yeast in the lung, aids immune system evasion [6]. Genitourinary blastomycosis, documented by Eickenberg et al. and Mouzin and Beilke, can arise through secondary infection or sexual contact, demanding repeated fungal cultures for confirmation [7-8]. Itraconazole is the preferred antifungal for both pulmonary and extrapulmonary cases, excluding meningitis [8]. This report details a rare case of uterine blastomycosis in a 51-year-old female presenting with pain and abnormal vaginal bleeding. In this context, the study aims to contribute insights into the unique manifestation of uterine involvement, addressing a significant gap in the current understanding of blastomycosis.

## Case presentation

We present the case of a 51-year-old female, a current cigarette smoker with a 40-pack-year history, who presented with abdominal, pelvic, and back pain and irregular vaginal bleeding for the past week.

**Figure 1:**
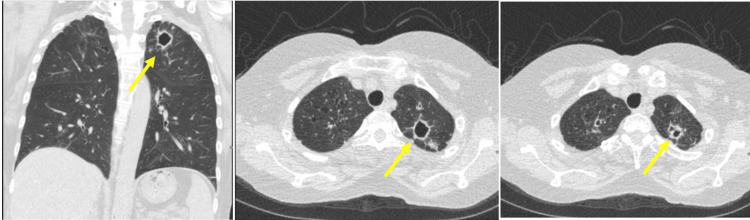
A lung CT scan showed multiple cavitary and solid nodules, including new ground glass opacities, observed bilaterally in lung apices with no mediastinal involvement (yellow arrow) CT: computed tomography

During the physical examination, abdominal and pelvic tenderness were noted, while the patient's vital signs remained within normal limits. The recent lung CT scan unveiled several concerning observations. Multiple cavitary lesions, particularly a notable enlargement in the left lung apex from its prior size, measured approximately 2.2 x 2.2 x 2.4 cm, contrasting with its earlier dimensions of 9.7 x 8.5 x 2.0 mm. Other findings included a solid noncalcified nodule in the right upper lobe measuring around 8 mm, a 10 mm cavitary nodule in the right lower lobe showing minimal change, and a new ground glass opacity nodule in the posterior left upper lobe, measuring approximately 2.3 x 1.7 x 1.9 cm, accompanied by smaller nodules in the same region and the lingula. Additionally, a nodular opacity within the bronchus intermedius, measuring around 6 x 4 x 4 mm, was identified, as shown in Figure 1.

**Figure 2:**
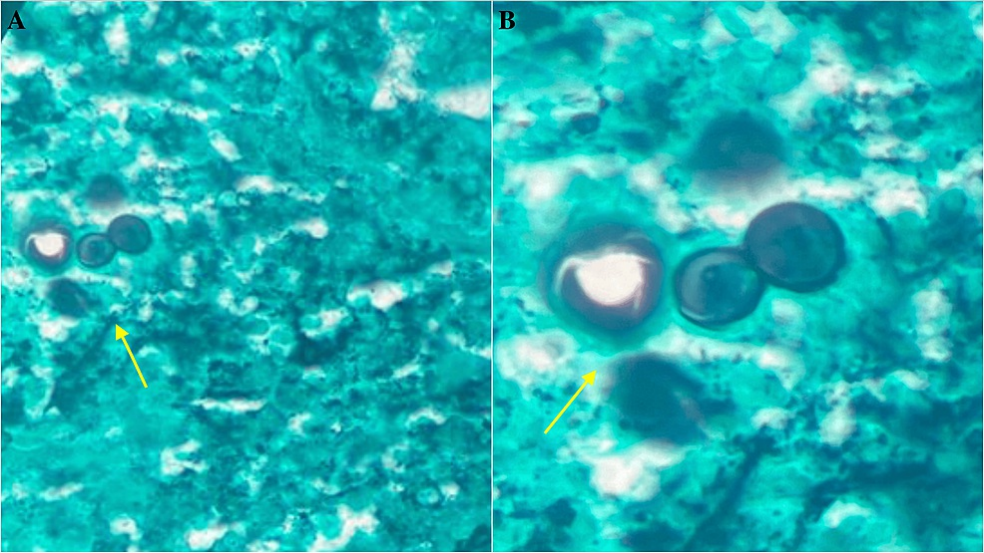
Gomori methenamine silver stain shows multiple areas of granulomatous endometritis with fungal forms consistent with blastomycosis (a) and broad budding yeast (b)

The American College of Radiology Lung Reporting and Data System flagged a potential lung inflammatory process, prompting a low-dose chest CT for further assessment. Consulting an infectious diseases specialist led to the diagnosis of uterine blastomycosis after an abnormal ultrasound. A subsequent hysteroscopic exam and curettage revealed necrotizing granulomatous endometritis. Pathology results identified fungal forms consistent with blastomycosis, confirmed via microscopic analysis showing broad budding yeast (Figure 2). Although initial tests were negative, immunohistochemical stains confirmed the presence of organisms. This detailed exploration ultimately confirmed the diagnosis of uterine blastomycosis.

The patient's management plan involved continuing the current oral itraconazole regimen of 200 mg twice daily initiated two weeks ago, with a follow-up scheduled in a month to assess treatment response and potential side effects. Long-term treatment aims for one year with itraconazole to ensure comprehensive management of the infection. Additionally, the patient was advised to consult her physician to discuss potential adjustments in the dose of Suboxone due to drug interactions between itraconazole, a P450 inhibitor, and Suboxone, which elevates buprenorphine-naloxone levels. Regular monitoring of clinical status, lung lesion improvement, adverse effects of itraconazole, and scheduling low-dose chest CT scans for monitoring are vital components of the management plan, emphasizing medication adherence, patient education, and coordinated care among healthcare providers for optimal outcomes. The patient responded well to the treatment given.

## Discussion

Blastomycosis typically presents as a pulmonary infection, which could slowly disseminate to produce extrapulmonary manifestations. Blastomycosis, in its acute and chronic form, may resemble other conditions in terms of symptomatology and imaging. Moreover, due to a large population being asymptomatic or developing subclinical infections, the disease often tends to go undiagnosed. The diagnosis of uterine blastomycosis is often challenging, owing to its non-specific complications [9]. Our patient presented with complaints of abdominal, pelvic, and back pain and irregular vaginal bleeding. These symptoms often mimic those of leiomyoma, endometriosis, adenomyosis, and pelvic inflammatory diseases, making distinguishing blastomycosis from more commonly encountered gynecological conditions difficult [10].

Genitourinary infection is the fourth most common manifestation of blastomycosis, following the lungs, skin, and bones [8]. However, to the best of our knowledge, there are fewer than 10 published case reports on uterine blastomycosis. The cases of blastomycosis documented in the female genital tract include regions such as the vulva, vagina, and uterus [11]. Female genital blastomycosis can be acquired either by hematogenous dissemination from a primary pulmonary focus or through sexual contact with a male infected with genitourinary blastomycosis. However, Mouzin and Beilke reported a case of a 28-year-old female with genital blastomycosis who had no historical or radiological evidence of primary pulmonary infection and had a non-infected sexual partner [8]. The most likely cause mentioned in the report is a remote, asymptomatic pulmonary focus.

It is also important to assess risk factors like exposure to soil, wood, or organic debris contaminated with the fungus and immunocompromised states such as HIV/AIDS and pregnancy. Gathering the patient’s travel history, particularly to endemic areas, is crucial for diagnosis and understanding potential routes of exposure. This aids in revealing geographical risk factors and directing appropriate approaches to clinical management. Diagnostic modalities for blastomycosis infections include chest radiography to demonstrate a primary focus of infection, a CT scan, histopathological examination, Grocott-Gomori methenamine silver nitrate staining, and fungal culture [8-9].

The effective management of a case of uterine blastomycosis would involve a multidisciplinary approach and a diverse team of gynecologists, pathologists, and infectious diseases specialists. Treatment for blastomycosis involves pharmacotherapy using antifungal agents like itraconazole and voriconazole after conducting a baseline laboratory examination and assessing hepatic and renal functions [3]. A clinical trial by Dismukes et al. on the efficacy and toxicity of orally administered itraconazole revealed that among 48 patients with non-meningeal blastomycosis infection, the total success rate of itraconazole was 90%. Among 40 patients who received at least two months of itraconazole therapy, 95% were cured of the infection [12]. Hence, prolonged treatment strategies lasting more than two months (preferably six months) can significantly decrease the chances of relapse of the infection.

Uterine blastomycosis, being a rare subset of systemic blastomycosis, is often underdiagnosed and underreported due to its clinical resemblance to more common gynecological conditions. Its non-specific symptoms further add to the complexity of its identification in routine clinical practice. The rarity of the condition and the scarcity of relevant literature pose major challenges in the development of standardized protocols for its diagnosis and treatment. Hence, there is a need for further research to better understand the pathogenesis and sequelae of this condition, thereby helping in devising effective diagnostic and treatment protocols tailored to it.

## Conclusions

Uterine blastomycosis poses diagnostic challenges due to its similarity to common gynecological conditions, necessitating comprehensive diagnostic approaches with imaging and fungal cultures. Its rarity highlights the need for heightened clinical suspicion, multidisciplinary management, and extended antifungal therapy. Further research is crucial to refine diagnostic and treatment protocols. Advancing our understanding will improve patient care. This case underscores the importance of expanding the knowledge base to refine diagnostic tools and optimize treatment strategies for uterine blastomycosis, potentially reshaping clinical approaches and enhancing patient outcomes in the future.
